# Healthcare utilization for somatic conditions among Swedish patients in opioid substitution treatment, with and without on-site primary healthcare

**DOI:** 10.1186/s12913-022-08351-1

**Published:** 2022-07-29

**Authors:** Teodor Vikbladh, Katja Troberg, Anders Håkansson, Disa Dahlman

**Affiliations:** 1 Center for Primary Health Care Research, Department of Clinical Sciences, Clinical Research Center/CRC, Lund University/Region Skåne, Box 503, 22 Malmö, Sweden; 2 Malmö Addiction Centre, Skåne University Hospital, Malmö, Sweden; 3 Division of Psychiatry, Department of Clinical Sciences Lund, Lund University, Lund, Sweden

**Keywords:** Opioid Substitution Treatment, Health Services Accessibility, Sweden, Health Equity, Primary Health Care

## Abstract

**Background:**

Opioid substitution treatment (OST) populations are aging and have increased mortality and somatic morbidity compared to general populations internationally. While OST patients have poor self-rated physical health and unmet healthcare needs, documented healthcare utilization has been sparsely investigated. The aim of this study was to assess registered healthcare utilization for somatic conditions in a sample of Swedish OST patients, and compare healthcare utilization among OST patients with and without use of on-site primary healthcare (PHC).

**Methods:**

Patients in OST in Malmö, Sweden, were recruited for a survey study conducted in 2017–2018. Survey data were compared with comprehensive patient records from specialized and primary care during one year prior to study inclusion (total *n* = 190). All patient records were examined for healthcare utilization, source of healthcare (PHC, emergency care and secondary care), and documented diagnoses and symptoms. Factors associated with healthcare utilization were analyzed by using logistic regression analysis. Patients with and without on-site PHC were compared by using descriptive statistics and Chi-2 test.

**Results:**

A total of 88% of the sample had been in direct or indirect contact with somatic healthcare during one year (PHC 66%; emergency care 28%; secondary care 67%). The most prevalent somatic diagnoses were infectious diseases (39%) and symptom diagnoses (37%). Respiratory, dermatological and musculoskeletal diagnoses, and trauma/intoxication were documented in 21–26% of the sample, respectively. PHC utilization was associated with older age and being born in Sweden. Among patients with on-site PHC (*n* = 25), the number utilizing secondary care was 84%, and certain diagnostic codes were more frequent in this group.

**Conclusion:**

OST patients are seemingly underserved as regards their physical health. Since increased OST access decreases opioid overdose fatalities, the life expectancy among OST patients is likely to increase and thereby also increases the risk of age-related conditions. Thus, easily accessible physical healthcare is of great importance in this group. On-site PHC might be a way to establish healthcare contact with OST patients, especially for non-acute conditions, although further research is needed.

## Background

Opioid substitution treatment (OST) effectively decreases overdose mortality and morbidity among people with opioid use disorder (OUD) [1, 2] and thereby increases the estimated life expectancy among patients in treatment. However, increasing age among OST populations is suspected to lead to increased medical comorbidities and cognitive impairment [3–5]. It is therefore of great importance that patients in OST have access to effective somatic healthcare.

A growing body of research shows that patients in OST are disproportionately affected by physical comorbidities. Drug-related as well as non-drug-related deaths are over-represented among OST patients compared to the general population [6, 7]. Some suggested reasons are circulatory diseases in combination with potent substitution medication [6, 8], cancers, respiratory diseases and digestive diseases [6]. Studies from Australia, Spain, the U.K. and the U.S. have identified electrocardiogram abnormalities, chronic obstructive pulmonary disease (COPD), asthma and liver disease, metabolic syndrome, diabetes, hypercholesterolemia and high blood pressure as frequent health issues among patients in OST [9–13]. Previous retrospective cohort studies have also shown high rates of chronic diseases, multimorbidity and high disease severity among patients receiving OST in primary healthcare (PHC) [14, 15] and high self-reported rates of geriatric conditions and multimorbidity [16]. Arnold-Reed et al. [14] showed that the prevalence of multimorbidity was almost 90% in patients receiving OST at a PHC center (Odds Ratio [OR] 7.29 compared to patients without OUD). In a study by O’Toole et al. [15], OST patients had significantly more chronic diseases (OR 9.1) and multimorbidity (OR 6.6) than other patients.

The high percentage of tobacco smoking (75% to 94% in previous studies) among OST patients [12, 17, 18] suggests that chronic respiratory and circulatory diseases are likely to be over-represented in this patient group. In addition, side effects from opioids suggest that patients in OST might have a high prevalence of constipation, sleeping apnea and sexual dysfunction/hypogonadism. Previous research from Scandinavia and North America has also shown that patients in OST have poor self-rated health and a heavy burden of physical symptoms [19–21]. Medved et al. [20] showed that almost three-quarters of Norwegian OST patients reported at least one chronic somatic condition, with hepatitis C (53%) and asthma (21%) being the most frequently reported.

In contrast to the findings indicating poor physical health and great healthcare needs among OST patients, a few previous studies have shown that patients in OST commonly have self-reported unmet healthcare needs and reluctance towards healthcare seeking [19, 22]. A recent study from Malmö, Sweden, showed low rates of circulatory diagnoses among OST patients, which likely indicates underdiagnosing in this patient group [23]. Spithoff et al. showed that patients in OST were less likely to receive diabetes monitoring than matched controls [24].

In 2016 an intervention was implemented in Malmö, Sweden, with the purpose of increasing access to PHC among OST patients by offering on-site appointment with a PHC physician at the OST clinic. This small-scale intervention, developed on the initiative from OST and PHC staff since 2014 with the aim to decrease healthcare barriers for OST patients, is described in greater detail by Bäckström et al. [23].

Several studies, mainly from Australia and North America, have stressed that people in OST and with active drug use receive fragmented medical care [25] and utilize emergency care rather than primary or outpatient care; this indicates a lack of long-term healthcare contacts necessary for effective treatment and prevention of chronic somatic conditions [26, 27]. A recent meta-analysis of 25 studies on emergency department (ED) episodes and 25 studies on hospital admissions among people who use illicit drugs showed pooled rates exceeding the general population rates by factors 4.8 and 7.1, respectively [27]. Frequent ED visits and inpatient episodes in this group are costly [28, 29] and suggested to be ineffective for meeting the high healthcare needs among people with substance use disorders (SUD) [27, 30–32].

Utilization of PHC has been subject to a lesser amount of research, and previous studies have shown heterogeneous results [27]. The care of patients receiving OST with methadone in PHC generated significantly more investigations, referrals, ED visits, outpatient attendances and hospital admissions than non-OST patients’ care [15]. In a retrospective cohort study from the U.S., regular primary medical care was associated with less hospitalization, among adults with SUD regarding illicit drugs [33].

Previous research on unmet healthcare needs has mostly been based on self-reports, and studies on registered healthcare utilization in general and PHC in particular [27] among OST patients are limited. We are not aware of any previous studies on documented healthcare utilization for somatic conditions among OST patients in a Scandinavian or similar context, where healthcare is comprehensive, tax-financed and strongly subsidized for the individual; thus, in a system where high health care access is theoretically facilitated, but where barriers may hypothetically remain among individuals with OUD.

The aim of this study was to assess registered healthcare utilization for somatic conditions in a sample of Swedish OST patients. A secondary aim was to compare healthcare utilization among OST patients, with and without use of on-site PHC, in order to generate hypotheses for future, larger studies.

## Methods

This was a retrospective study based on medical records and self-reported data from OST patients in Malmö, Sweden. The study has been approved by the Regional Ethics Board, Lund, Sweden, file no. 2016/1105.

### Setting

The study was conducted in Malmö, Sweden. With approximately 350,000 residents, Malmö is the third largest city in Sweden. It is located in Skåne county in southern Sweden.

In Sweden, a country with high opioid overdose mortality [34] and traditionally restrictive drug policy [35], physical healthcare as well as OST is covered by the universal health insurance and tax-financed [36], which makes the healthcare service strongly subsidized for the individual. There are public as well as private healthcare providers.

Acute somatic conditions are treated at PHC level or at emergency departments (depending on severity), while non-acute and chronic conditions are treated at PHCs or specialized secondary care clinics. Primary healthcare in Sweden is comprehensive, and all Swedish citizens and permanent residents with a personal number are automatically registered at a PHC center (which can be actively changed by the individual). In general, specialized secondary clinics accept new patients only after a referral from another caregiver – typically a PHC physician. In Malmö, there is a large university hospital providing comprehensive emergency, inpatient and outpatient care.

In Sweden, OST is provided at specialized psychiatric treatment units that can be run by public as well as private caregivers. People with OUD are eligible for OST if they are considered to have had OUD for at least one year, and are generally 20 years of age or older. OST in Sweden includes psychosocial/psychological treatment and repeated testing for blood-borne infections, in addition to the pharmacological treatment with buprenorphine or methadone. In Sweden, an estimated 4,000 individuals (at least) receive OST, but OST availability varies depending on geographical region [37]. With 24 OST clinics in a population of 1.4 million, Skåne county has high availability for this treatment. By the time that this study was conducted, there were five OST clinics in Malmö providing treatment to approximately 520 patients with OUD.

By the time this study was conducted, patients at two OST clinics in Malmö (both included in the study) were offered on-site appointments every second week with a PHC physician from one designated PHC center.

### Participants

Patients from four of the total five OST clinics (three public and one private clinic) in Malmö, Sweden, were recruited to a survey study about self-rated health and healthcare seeking in 2017–2018, as described by Troberg et al. [19]. The intent was to offer every patient participation in the study. By providing informed consent to study participation, the individuals also gave consent to a subsequent patient record analysis one year prior to inclusion in the survey study. The only inclusion criterion was receiving OST in any of the four OST clinics in the study. Exclusion criteria were severe psychiatric conditions, language barriers or drug influence hindering the individual from giving informed consent. No monetary compensation was involved for participating in the study.

### Procedures and data management

#### Survey data

Data regarding age, sex, country of birth, main source of income, housing status, smoking habits, and physical symptoms (respiratory, gastrointestinal, genitourinary/sexual problems, pain from extremities, neck or back) were collected through self-reports via a survey that was distributed at four OST clinics, as described by Troberg et al. [19].

Prior to analysis, three variables, with multiple choice answers in the survey, were recoded: Housing situating was recoded to “unstable housing” if the respondent replied “transitional apartment”, “institution/family care placement”, “hotel”, “homeless” or “other”. Main source of income was dichotomized into “employment” (multiple choice alternative) and “other income” (if the respondent replied “public assistance”, “old age pension”, “sick leave”, “permanent sick leave” or “other”). Smoking habits were recoded to “current smoker” if the answer was “smoke daily” or “smoke less than daily”.

Self-reported physical symptoms and worries about physical health were recoded as described by Troberg et al. [19]: If a participant had described symptoms/worries in the open-end question but data were missing for yes/no questions regarding specific symptoms/worries, the answer was recoded to “yes”.

#### Patient record data

Medical records from primary care and emergency/secondary care in Skåne county for all survey study participants (*n* = 218) were retrieved for one year prior to study participation for each individual. Skåne county has two separate, digital patient record systems; one for primary care and one for emergency and secondary care (inpatient and outpatient clinics). Both public and private caregivers use these two patient record systems.

Patient records from emergency and secondary care were read digitally, while primary care records were retrieved as paper copies. All individuals for whom access to *both* primary and emergency/secondary care records was not allowed (due to confidentiality requested by the patient or invalid ID) were excluded from further analysis. Of the 218 individuals (72% male; median age 43 years; 76% born in Sweden) who gave informed consent to participate in the survey study, 28 (79% male; median age 45 years; 86% born in Sweden) were excluded, leaving 190 individuals for patient record analysis.

Each patient record was read manually by one or two of the authors (T.V. and D.D., both medical doctors), and searched for the following variables:

##### Sources of healthcare

All contacts, both visits and indirect contacts (e.g., no-shows, telephone appointments, written responses to referrals, patient leaving before examination), were noted. Psychiatric reasons for healthcare utilization were not included in the analyzes. No restrictions regarding healthcare personnel were applied, but we included healthcare contacts documented by, e.g., physicians, nurses, physiotherapists, psychologists, dieticians and occupational therapists.

*Primary care utilization* was defined as any registered contact for non-psychiatric conditions in the patient record system for PHC.

*Emergency/secondary care utilization* was defined as any registered contact in the patient record system for emergency/secondary care (in- and outpatient), excluding psychiatric care, dementia care, and SUD care. Emergency care was defined as contact with the emergency room or emergency ward. The specific secondary clinics where the individual had registered contacts were noted. In some cases, patients had registered healthcare contact with private clinics with inaccessible patient record documentation. These contacts were noted as “unclear”.

##### Physical conditions noted in the patient records

All patient records were searched for diagnosis codes; symptoms and conditions described in plain text; and other contacts indicating a specific problem (e.g. renewal of a prescription). The conditions and symptoms were clustered in accordance with ICD-10 diagnostic codes, with the purpose of presenting the results in a way that has relevance from a clinical perspective. Symptom diagnoses (categories under the R chapter in ICD-10) were included only if they were not coherent with another somatic diagnosis. Non-psychiatric contact reasons not covered by ICD-10 diagnostic codes were recoded as “other”. Uncertain diagnoses (e.g. patient record notifications of received referrals without a specified cause) were noted as “unclear”.

We also searched all patient records for the following chronic somatic conditions (noted as ICD-10 codes or plain text), and noted the source of healthcare (primary/emergency/secondary care) where the diagnosis was obtained: Hepatitis C (ICD-10 B18.2-, with and without fibrosis/cirrhosis); asthma (ICD-10 J45-); COPD (ICD-10 J44-); diabetes (ICD-10 E10-, E11-, E14-); hypogonadism in males (ICD-10 E29.1) and constipation (ICD-10 K59.0). These conditions were selected in compliance with side effects from OST medications, and in order to correspond to specific diagnoses assessed in previous research. Data on documented cardiovascular diseases has been previously reported [23] and was thus not included here.

In addition, we noted if the patient had been offered or received any intervention for smoking cessation, such as a prescription for varenicline or a motivational interview.

##### On-site primary healthcare in OST

Utilization of on-site PHC in OST was defined as any notes in the PHC records that the patient had had an appointment with a PHC physician at the OST clinic. All individuals who had not utilized on-site PHC were defined as having regular PHC, regardless of whether they had been in contact with PHC or not.

### Analysis

The collected data were organized in SPSS Statistics Version 25 [38] and analyzed by using descriptive statistics. Factors associated with primary, emergency and secondary care utilization (direct or indirect contact) were analyzed by unadjusted and adjusted logistic regression analysis. The covariates were socioeconomic variables, self-reported symptoms and self-reported worries about one’s physical health. Due to the limited sample size, ICD diagnoses noted in the patient records were not included in the analyses. We adjusted the analyzes for age, sex, and exposures significantly associated with the outcome variable in univariate analysis.

The data of the group of patients who had utilized on-site PHC was compared with the group of patients without on-site PHC by using descriptive statistics and Chi-2 test (Fisher’s exact test for variables with low cell counts). We analyzed potential associations between on-site PHC and source of healthcare utilization (contacts and visits, respectively). *P* < 0.05 was considered statistically significant.

## Results

### Sample characteristics

Of the 190 participants, 25 (64% male, median age 50 years) had utilized on-site PHC and 165 (72% male, median age 42 years) had utilized regular or no PHC (Table 1). Unstable housing was reported by 44% of those utilizing on-site PHC, and by 22% of those who did not utilize on-site PHC. More than 80% reported current tobacco smoking. As previously reported by Troberg et. al. [19], self-reported physical symptoms and worries about one’s physical health were common. The sub-sample utilizing on-site PHC (*n* = 25) had significantly more often unstable housing situations (*p* = 0.004) and worries about their physical health (*p* = 0.034) than individuals utilizing regular PHC (Table 1). We found no associations between on-site PHC utilization and age, sex, country of birth, employment, tobacco smoking, or self-reported physical symptoms.

**Table 1:** Self-reported sample characteristics among patients in OST with regular PHC and on-site PHC. Chi-2 test, Fisher’s exact test or t-test. *N* = 190

Characteristic	Valid n	Regular PHC	On-site PHC	*p*-value
		**n (%) or years (range; IQR or SD)**^a^	**n (%) or years (range; IQR or SD)**^a^	
Total N	190	165	25	
Median age	190	42 (23–65; 37–51)	50 (25–63; 36–54)	
Mean age	190	44 (23–65; 10.0)	46 (25–63; 10.6)	0.375^b^
Male sex	190	119 (72)	16 (64)	0.404
Born in Sweden	188	125 (76)	16 (64)	0.173
Unstable housing	187	30 (18)	11 (44)	0.004*
Employment as main source of income	184	29 (18)	3 (12)	0.770^c^
Current tobacco smoking	179	134 (81)	21 (84)	1.000^c^
Respiratory symptoms	178	74 (45)	12 (48)	0.532
Gastrointestinal symptoms	179	86 (52)	14 (56)	0.794
Genital or sexual symptoms	180	72 (44)	14 (56)	0.266
Pain from extremities, back or neck	182	101 (61)	17 (68)	0.329
Worries about physical health	178	85 (52)	18 (72)	0.034*

*OST* Opioid substitution treatment, *IQR* Interquartile range (for median numbers), *PHC* Primary healthcare, *SD* Standard deviation (for mean numbers)

^a^Missing numbers are included in the denominator. b. T-test. c. Fisher’s exact test (for variables with low cell counts)

^*^ *p* < 0.05

### Healthcare utilization for somatic conditions

#### Sources of healthcare

A total of 88% of the sample had been in direct or indirect contact with any kind of somatic healthcare during the year preceding study inclusion (Table 2). Two thirds of the sample had been in contact with PHC (66%) or specialized secondary care (67%; mainly the Infectious diseases clinic). Emergency care contacts were registered in 28% of the sample, and 10% of the sample had received inpatient somatic care. Physical visits to PHC were documented in 57%, to emergency care in 25%, and to secondary care in 58%.

**Table 2:** Physical conditions among OST patients, noted in patient records. *N* = 190

**Characteristic**	**Any healthcare** n (%)	**Primary care**^**a**^ n (%)	**Emergency care**^**a**^ n (%)	**Secondary care**^**a**^ n (%)
Contact for any reason	168 (88)^1^	126 (66)	53 (28)	127 (67)^2^
Visit for any reason	160 (84)^2^	108 (57)	48 (25)	110 (58)^3^
Infectious diseases (ICD-10 A00-B99)	74 (39)^b^	41 (22)	3 (2)	57 (30)
Tumors (ICD-10 C00-D48)	7 (4)	4 (2)	0	4 (2)
Hematologic diseases (ICD-10 D50-D89)	9 (5)	9 (5)	0	1 (< 1)
Endocrine diseases (ICD-10 E00-E90)	30 (16)^c^	22 (12)	0	14 (7)
Neurological diseases (ICD-10 G00-G99)	20 (11)	16 (8)	4 (2)	8 (4)
Eye diseases (ICD-10 H00-H59)	7 (4)	6 (3)	0	1 (< 1)
Ear diseases (ICD-10 H60-H95)	7 (4)	7 (4)	0	1 (< 1)
Circulatory diseases (ICD-10 I00-I99)	26 (14)	23 (12)	1 (< 1)	3 (2)
Respiratory diseases (ICD-10 J00-J99)	39 (21)^d^	37 (20)	3 (2)	2 (1)
Gastrointestinal diseases (ICD-10 K00-K93)	33 (17)^e^	25 (13)	0	15 (8)
Dermatologic diseases (ICD-10 L00-L99)	44 (23)	32 (17)	2 (1)	19 (10)
Musculoskeletal diseases (ICD-10 M00-M99)	49 (26)	41 (22)	10 (5)	14 (7)
Urogenital diseases (ICD-10 N00-N99)	20 (11)	15 (8)	0	6 (3)
Obstetrics (ICD-10 O00-O99)	2 (1)	0	0	2 (1)
Symptoms (ICD-10 R00-R99)	70 (37)	60 (32)	15 (8)	10 (5)
Trauma, poisoning etc. (ICD-10 S00-T98)	46 (24)	24 (13)	30 (16)	10 (5)
Other^f^	29 (15)	19 (10)	0	14 (7)
*Smoking cessation*	*10 (5)*	*10 (5)*	*0*	*0*
Not specified^g^	16 (8)	0	4 (2)	12 (6)

^1^Missing *n* = 1

^2^Missing *n* = 2

^3^Missing *n* = 4

^a^Not mutually exclusive. Emergency care includes emergency room and emergency ward

^b^Hepatitis C (B18.2-): *n* = 67 (35%)

^c^Diabetes (E10-, E11-, E14-): *n* = 6 (3%). Hypogonadism in males (E29.1): *n* = 11 (6%)

^d^Asthma (J45-): *n* = 13 (7%). Chronic obstructive pulmonary disease (J44-): *n* = 13 (7%)

^e^Constipation (K59.0): *n* = 11 (6%)

^f^Smoking cessation, unspecified allergy (T78.4), sexual dysfunction (F52-), STD testing, contraceptives, pap smear, vaccine, research participation

^g^Unclear diagnosis due to patient leaving prior to reporting one’s symptoms/examination, or due to no-shows to secondary outpatient care and unclear contact reason/unclear reason for referral

In univariate analysis, PHC utilization was associated with higher age (OR 1.04; 95% CI 1.01–1.08; *p* = 0.007) and with being born in Sweden (OR 2.23; 95% CI 1.02–4.85; *p* = 0.043), but not with sex, employment, housing status, smoking, self-reported physical symptoms or self-reported worries about one’s physical health (Table 3). In a multivariate analysis including age, sex and country of birth as covariates, these associations remained statistically significant for age (Adjusted Odds ratio [AOR] 1.05; 95% CI 1.02–1.08; *p* = 0.007) and being born in Sweden (AOR 2.34; 95% CI 1.05–5.19; *p* = 0.037). We did not find any associations with utilization of somatic healthcare (primary/emergency/secondary), emergency care, or secondary care (numbers not shown in the manuscript).

**Table 3:** Factors associated with primary healthcare utilization among patients in OST. Univariate and multivariate logistic regression. *N* = 190

Covariates	Univariate analysis			Multivariate analysis		
	**Valid n**	**OR (95% CI)**	***p*-value**	**Valid n**	**AOR (95% CI)**	***p*-value**
Median age in years	190	1.04 (1.01–1.08)*	0.007*	188	1.05 (1.02–1.08)*	0.007*
Male sex	190	0.74 (0.38–1.47)	0.393	188	0.64 (0.32–1.31)	0.222
Born in Sweden	188	2.23 (1.02–4.85)*	0.043*	188	2.34 (1.05–5.19)*	0.037*
Unstable housing	187	1.30 (0.59–2.68)	0.550		N/A^a^	N/A
Main income through employment	184	0.59 (0.27–1.29)	0.188		N/A^a^	N/A
Current tobacco smoking	179	1.02 (0.41–2.54)	0.967		N/A^a^	N/A
Respiratory symptoms	178	1.74 (0.93–3.26)	0.085		N/A^a^	N/A
Gastrointestinal symptoms	179	0.82 (0.44–1.54)	0.542		N/A^a^	N/A
Genital or sexual symptoms	180	0.79 (0.43–1.47)	0.460		N/A^a^	N/A
Pain from extremities, back or neck	182	1.26 (0.67–2.39)	0.472		N/A^a^	N/A
Worries about physical health	178	1.62 (0.87–3.04)	0.131		N/A^a^	N/A
On-site PHC utilization	190	N/A	N/A		N/A	N/A
OST clinic with on-site PHC	190	1.00 (0.54–1.83)	0.987		N/A^a^	N/A

*OST* Opioid substitution treatment, *PHC* Primary healthcare, *OR* Odds ratio, *AOR* Adjusted Odds ratio, *CI* Confidence interval, *N/A* Not applicable

^*^ *p* < 0.05

^a^Variable not included in the multivariable analysis due to lack of association with the outcome variable in univariate analysis

#### Somatic diagnoses noted in the patient records

The most frequently documented physical conditions were infectious diseases (39%) and symptom diagnoses (37%). Musculoskeletal diagnoses, trauma/poisoning, dermatological diagnoses and respiratory diagnoses were registered in 21–26% of the sample. All other somatic diagnostic groups were less frequently registered, in 1–17% (Table 2).

With the exception of hepatitis C, which was noted in 35% of the sample, specific chronic somatic diagnoses were surprisingly infrequent. Asthma and COPD were noted in 7% of the sample, respectively; hypogonadism and constipation in 6%, respectively; and diabetes in 3%. Ten individuals (5%) had been offered help with smoking cessation.

The most frequent diagnoses in PHC were symptom diagnoses (32% of the sample) followed by infectious diseases and musculoskeletal diagnoses. In emergency care, the most frequent diagnoses were trauma/poisoning (16% of the sample), symptom diagnoses and musculoskeletal diagnoses. In secondary care, infectious diseases (30% of the sample), dermatologic diagnoses and gastrointestinal diagnoses were the most prevalent diagnoses.

### Healthcare utilization among patients with on-site primary care

Primary care utilization was by definition registered for all individuals with on-site PHC, while 61% of the sub-sample without this intervention had been in direct or indirect contact with PHC (*p* < 0.001). Healthcare utilization by a physical visit (primary/secondary/emergency) was also associated with on-site PHC (*p* = 0.029). A total of 50% of the sample with regular PHC had been to a physical PHC visit for a somatic condition. We found no statistically significant differences in emergency care and secondary care utilization between the two groups. However, notably more patients with on-site PHC (84%) than regular PHC (64%) had been in direct or indirect contact with secondary care. (Table 4).

**Table 4:** Healthcare utilization and registered chronic somatic diagnoses in OST patients with regular PHC and on-site PHC. *N* = 190. Chi-2 test or Fisher’s exact test

	**N included in analysis**^a^	**Regular PHC** n (%)	**On-site PHC** n (%)	***p*-value**
Total N	190	165	25	
Any healthcare contact	189	143 (87)	25 (100)	0.082^b^
Any healthcare visit	188	135 (82)	25 (100)	0.029*^b^
Primary care contact	190	101 (61)	25 (100)	< 0.001*^b^
Primary care visit	190	83 (50)	25 (100)	< 0.001*^b^
Emergency care contact	190	49 (30)	8 (32)	0.815
Emergency care visit	190	45 (27)	7 (28)	0.939
Secondary care contact	188	105 (64)	21 (84)	0.052
Secondary care visit	186	94 (57)	15 (60)	0.491
Infectious diseases (ICD-10 A00-B99)	190	55 (33)	19 (76)	< 0.001*
Tumors (ICD-10 C00-D48)	190	5 (3)	2 (8)	0.231^b^
Hematologic diseases (ICD-10 D50-D89)	190	9 (6)	0	0.609^b^
Endocrine diseases (ICD-10 E00-E90)	190	23 (14)	7 (28)	0.082^b^
Eye diseases (ICD-10 H00-H59)	190	5 (3)	2 (8)	0.231^b^
Ear diseases (ICD-10 H60-H95)	190	4 (2)	3 (12)	0.050*^b^
Neurological diseases (ICD-10 G00-G99)	190	13 (8)	7 (28)	0.007*^b^
Circulatory diseases (ICD-10 I00-I99)	190	23 (14)	3 (12)	1.000^b^
Respiratory diseases (ICD-10 J00-J99)	190	32 (19)	6 (24)	0.592
Gastrointestinal diseases (ICD-10 K00-K93)	190	25 (15)	8 (32)	0.049*^b^
Dermatologic diseases (ICD-10 L00-L99)	190	34 (21)	11 (44)	0.010*
Musculoskeletal diseases (ICD-10 M00-M99)	190	36 (22)	13 (52)	0.001*
Urogenital diseases (ICD-10 N00-N99)	190	17 (10)	3 (12)	0.732^b^
Obstetrics (ICD-10 O00-O99)	190	2 (1)	0	1.000^b^
Symptoms (ICD-10 R00-R99)	190	51 (31)	19 (76)	< 0.001*
Trauma, poisoning etc. (ICD-10 S00-T98)	190	37 (22)	9 (36)	0.140

*OST* Opioid substitution treatment, *PHC* Primary healthcare

a. Missing numbers are included in the denominator. b. Fisher’s exact test (for variables with low cell counts)

^*^ *p* < 0.05

The most common registered diagnostic clusters in the on-site PHC group were infectious diseases and symptom diagnoses (76%, respectively; Table 4). More than half of the sub-sample (52%) had musculoskeletal diagnoses. Gastrointestinal diagnoses, dermatological diagnoses and trauma/poisoning were registered in 32–44%; and respiratory, neurological and endocrine diagnoses in 24–28% of the sub-sample. All other somatic diagnostic groups were less frequently registered, in 0–12%.

## Discussion

This study, which is one of the first to examine somatic healthcare utilization among Scandinavian patients in OST, showed that a majority (88%) had been in direct or indirect contact with somatic healthcare during one year. Primary and specialized secondary care was utilized by two-thirds of the sample, and emergency care by a third of the sample. However, a notable quota of the healthcare contacts never led to a physical visit (e.g. no-shows after a referral was sent or a PHC appointment was made by OST staff).

Almost 40% of patients without on-site PHC did not have any contact with PHC during one year, and in this sub-sample only 50% had been to a PHC visit. Although data on the percentage of PHC contacts in the general Swedish population are not available, the numbers in our study are low in comparison with local statistics from another region in Southern Sweden. Previous Swedish data reported a 71% frequency of contact with PHC during a year overall in a community sample, and 70% for individuals aged 25–44, which is somewhat younger than the OST average [39]. In 2019, there were 13,257,132 registered visits to a PHC physician [40] in the total Swedish population of 10,319,473 [41], indicating that PHC is frequently utilized by the Swedish population. The numbers in our study are also notably lower than the self-reported PHC utilization in a recent Norwegian study. Medved et. al. [20] showed that 81% of long-term OST patients reported that they had utilized PHC during the past six months, and 51% reported that they had utilized other healthcare than PHC during the same period. In a study by Saitz et al. [42], which assessed factors associated with PHC, 41% of almost 6,000 people in addiction treatment reported that they did not have a PHC physician. The numbers shown by Saitz et al. are similar to those in our study, although their study was conducted in the 1990s U.S.

Emergency care contacts were common (28%) in our sample, which is similar to previous data on patients receiving OST with methadone in the U.S. (24% and 33% among patients with and without on-site medical care, respectively) [25], and higher than the 10% of OST patients visiting an ED in the past 12 months, reported by Clay et al. [43]. A comparison between Swedish and U.S. data is difficult to make because of the large differences in the countries’ healthcare systems. However, our numbers are higher than in the general Swedish population. In 2017, there were 225,288 ED visits by individuals aged 19 and older in Skåne [44], with a population of 1,044,783 in the same year and age span [41]. Since each individual may have several visits, a maximum of 22% of the general population in Skåne had utilized emergency care during one year. In the general Swedish population, elderly people aged 80 and older account for almost 20% of ED visits [45], which is in contrast to our study sample where the median age was only 43 years and no one was over 65 years old. Reasons for ED rather than other healthcare utilization from the scientific literature have been suggested to be, e.g., trust issues and fear of stigma [46], psychosocial difficulties and psychiatric comorbidities including periods of active drug use [47].

Specialized secondary care utilization was more prevalent than PHC utilization, which is surprising due to the structure of the Swedish healthcare system, where PHC is the first instance of care. The majority of individuals utilizing secondary care had been in contact with the Infectious diseases clinic (47% of the sample), which includes a needle exchange program (NEP) facility that serves as a PHC facility and does not require a referral. It is thus possible that the individuals in this study utilize the NEP, or already established contact at the Infectious diseases clinic, rather than a PHC. This hypothesis is supported by previous qualitative findings from Malmö, Sweden, where OST patients expressed reluctance towards healthcare contacts other than OST and NEP due to fear of stigmatizing treatment [46].

Somewhat surprisingly, we found no significant associations between emergency or secondary care utilization and demographic/socioeconomic variables, self-reported symptoms or self-reported worries about one’s physical health. This is in contrast to previous research consistently showing associations between ER presentation and female sex and homelessness [27]. Primary care utilization was associated only with higher age and being born in Sweden. Women had more PHC contact than men according to Swedish data on the general population [39], an association that was not found in our sample. Previous research on correlates of PHC utilization among OST patients is sparse. In an older study based on self-reports, lack of health insurance, male sex and younger age were associated with not having a PHC physician, while recent emergency‐room visits were not [42]. The relatively small numbers of women (*n* = 55), individuals born outside Sweden (*n* = 51), individuals with unstable housing (*n* = 41) and individuals who reported employment as their main source of income (*n* = 32) in our study imply that the negative results should be interpreted with caution.

Infectious diseases, symptom diagnoses, musculoskeletal conditions and trauma/poisoning were the most prevalent registered diagnostic clusters. Trauma and intoxication were the contact reason in half of the ED contacts in our study (14% of the sample). With the exception of hepatitis C, specific chronic somatic diagnoses were unfrequently registered. A recent review article showed that infections, traumas and injuries were common reasons for ED visits and inpatient episodes among people who use illicit drugs internationally [27].

Asthma and COPD were noted in 7% of the sample, respectively, which is notably lower than expected given the high percentage of tobacco smoking, and lower than the 21% with self-reported asthma in the recent study by Medved et al. [20]. In the general Swedish population, 9% reported daily tobacco smoking when the study was conducted [48], and the prevalence of COPD has been 6–16% in previous population-based studies [49, 50]. Islam et al. [10] detected COPD by physical examination in 30% of methadone patients in Australia. It is notable that respiratory conditions were prevalently diagnosed (21%) in the sample, but most of these diagnoses were acute conditions such as upper respiratory tract infection and pneumonia. In order to diagnose asthma and COPD, the patient needs to comply with examinations and follow-up, which might constitute a barrier to correct diagnosing. While over 80% reported tobacco smoking, only 5% had been offered any intervention for smoking cessation in physical healthcare. It has been shown that people with substance dependence in treatment perceive the associated risk of smoking as lower [51]. According to a review article from the U.S., most (76–80%) patients in OST with methadone have a desire to quit smoking, but only a minority receive assisted smoking cessation or referrals for smoking cessation intervention [17]. In a systematic review article, Apollonio et al. [52] concluded that “Overall, the results suggest that tobacco cessation interventions incorporating pharmacotherapy should be incorporated into clinical practice to reduce tobacco addiction among people in treatment for or recovery from alcohol and other drug dependence.”

Conditions related to opioid side effects (hypogonadism, constipation) were documented in 3–6% of the sample. These numbers are surprisingly low, and are unlikely to represent the actual burden of symptoms in the patient group. The prevalence of opioid-induced constipation was 60% among OST patients in previous studies [53]. To some extent, the patients might receive help from their OST clinics with opioid-related problems (e.g. prescriptions for treatment of constipation, impotence and hyperhidrosis). Patients might also normalize opioid side effects. While constipation can be diagnosed after a single healthcare appointment, diagnosing hypogonadism demands blood tests and follow-ups and is thus more dependent on patient compliance.

The small group of OST patients (*n* = 25) who utilized on-site PHC had notably higher prevalence of PHC contacts (100% vs. 61% among those utilizing PHC as usual) and secondary care contacts (84% vs. 64%). There was no difference between the groups regarding emergency care contacts (28% in both groups).

There is limited previous research on associations between on-site PHC in OST, and general and symptom-specific somatic healthcare utilization. However, older, international studies have shown positive results from linking SUD treatment (including but not limited to OST) with physical healthcare including PHC, regarding treatment of infectious and chronic diseases [54–57]. Gourevitch et al. [25] showed that OST patients receiving on-site medical care had significantly more outpatient visits, fewer hospitalizations and fewer emergency department visits. In a longitudinal study, Friedmann et al. [58] found that on-site PHC reduced subsequent ED and hospital use among patients in OST with methadone. We did not aim to analyze effects on ER or inpatient care by on-site PHC, due to small sample size and short time of data retrieval. The study design did not allow interpretations of causality, since the on-site PHC contacts may have been established at the end of the study period.

While the small number of individuals in the on-site PHC group motivates caution when interpreting the results, certain diagnostic clusters were notably more prevalent in the on-site PHC group. The analyzes in this study could not clarify whether patients utilizing on-site PHC were more burdened by specific diseases, or whether they had less unmet healthcare needs than those receiving regular PHC. However, we found no statistically significant associations between on-site PHC utilization and self-reported physical symptoms.

A possible reason for the higher number of certain diagnostic clusters in the on-site PHC group is that the patients’ OST status is disclosed to the on-site PHC physician, who tends to focus more on, and actively ask about, conditions related to opioid use and prior injection drug use. Infectious diseases—predominantly hepatitis C—were diagnosed in 76% of the on-site group and in 33% in the regular PHC group; and gastrointestinal diagnoses including constipation were registered in 32% in the on-site group and 15% in the regular PHC group. Long-term conditions might be normalized by the patients and not brought up spontaneously. On the other hand, patients in Australia in OST with methadone and buprenorphine reported that dental problems, constipation and headache were the most common reasons for healthcare seeking, while sweating (26%) and sexual dysfunction (24%) were reported as problems for which the participants would currently like help with [22].

Musculoskeletal diseases were diagnosed in 52% of the on-site sub-sample and 22% in the regular PHC sub-sample. This could partially be explained by the higher percentage with unstable housing in the on-site PHC group, since unstable housing was associated with self-reported pain from back, neck or extremities in the same sample [19]. Another potential explanation could be that patients in OST may avoid seeking healthcare for musculoskeletal pain, due to fear of stigma and so-called mutual mistrust [59]. Since 62% of our sample reported neck, back or extremity pain (no significant differences between patients receiving on-site PHC and others), and previous studies have shown that methadone patients report 80% prevalence of recent non-specified pain [60] and 37% prevalence of severe chronic pain [60, 61], we hypothesize underdiagnosing in the sub-group utilizing regular PHC.

In comparison with self-reported symptoms and previous research on physical conditions, OST patients seem to under-utilize healthcare and are seemingly underdiagnosed with regard to chronic conditions such as COPD. Approximately half the sample reported symptoms from the airways (45%), gastrointestinal system (53%), genitals (45%) and musculoskeletal pain (62%). Interestingly, we did not find any statistically significant associations between self-reported symptoms and healthcare utilization. This finding indicates that the high percentage of self-reported unmet healthcare needs presented by Troberg et al. [19] might be coherent with the patient record documentation. Future research assessing the accuracy of OST patients’ self-reports would add valuable findings to the literature.

Our results support previous findings that people with SUD and OST patients seek healthcare sporadically for acute symptoms rather than establishing long-term healthcare contacts for diagnosis and treatment of chronic conditions [25–27].

This study has limitations. The number of study participants, especially in the on-site PHC group, was relatively small, which makes it difficult to draw conclusions from the statistical analyzes. However, our study sample made up 37% of the total number of OST patients in Malmö by the time of study inclusion. The sample is considered fairly representative for the OST population in Malmö, as there were only small differences between our sample and the total OST population regarding sex distribution (male sex: 71% vs. 72%) and median age (43 years vs. 45 years).

The results may not be generalizable outside Skåne county, which is a Swedish region with high OST availability and comprehensive healthcare coverage. The city of Malmö has a university hospital and easily accessible emergency, secondary and primary care. Malmö also has a long tradition of harm reduction interventions, such as needle exchange and maternal care for women with SUD. The healthcare utilization in this study might therefore not be transferable to other regions in Sweden, with poorer OST and other healthcare access. The results should be interpreted with regard to that Swedish healthcare is strongly subsidized for the patients, and PHC is comprehensive. The results could therefore not be directly translated to other contexts.

In addition, patient record analysis involves a subjective judgment since we noted not only ICD-10 codes but also plain text. In order to secure a reading as objective as possible, the patient record data were analyzed by two of the authors (T.V. and D.D.), who are both medical doctors.

Our results may have important clinical implications. Since increased OST access decreases opioid overdose fatalities, the life expectancy among OST patients is likely to increase and thereby also increases the risk of age-related conditions. Thus, easily accessible physical healthcare is of great importance in this group. Previous studies have stressed the need for integrated physical and psychiatric care in order to meet the healthcare needs in the OST population [14] and aging people with SUD [62]. We suggest that integration of medical care in OST, such as on-site PHC or offering of yearly basic physical examinations, should be taken into consideration by healthcare workers and policy makers.

More importantly, the results from this study have research implications. Based on our findings, we suggest that future, large-scale studies could assess the following subjects:

1. Assessment of healthcare quality and continuity for OST patients. Patients in OST seem to under-utilize healthcare for physical conditions. Even though ER rates are high in our study as well as previous research, PHC utilization was low in our study. A significant quota of the healthcare contacts in our sample were no-shows. We therefore suggest that future research should assess not only the numbers of healthcare contacts, but also quality of care, follow-up of referrals and planned treatments, and continuity.
2. Physical morbidity assessed by anthropometrics. Patients in OST seem to be underdiagnosed with chronic physical conditions such as COPD, arthritis and cardiovascular diseases. Our results indicate that OST patients seek healthcare sporadically for obvious symptoms (e.g. dyspnea, trauma), while chronic, non-acute conditions (e.g. COPD, arthritis) tend to remain undiagnosed. We therefore suggest that physical morbidity among OST patients should be assessed by standardized anthropometrics rather than registered diagnoses or self-reports.
3. On-site PHC should be evaluated regarding the continuity and quality of care for OST patients. Future studies with larger sample size would be valuable to assess healthcare seeking patterns among OST patients with and without on-site PHC. In our study, on-site PHC was utilized by socioeconomically challenged OST patients with unstable housing situations, which might indicate that individuals utilizing this service have a greater disease and symptom burden than others. A comparison of self-rated or measured physical health in patients, with and without on-site PHC, might show poorer results in the on-site group, due to poorer health at baseline. Although our findings are tentative, we hypothesize that on-site PHC in a safe, non-stigmatizing environment might be particularly efficient to assess non-acute conditions that might be considered as complicated (e.g. musculoskeletal pain).

## Conclusions

This study is one of the first to assess healthcare utilization for physical symptoms/conditions among Swedish patients in OST, and showed that OST patients are seemingly underserved as regards their physical health. On-site PHC might be a way to establish a healthcare contact with OST patients, especially for non-acute conditions that might be considered sensitive, although further research is needed.

## Data Availability

The SPSS data used to support the findings of this study are restricted by the Regional Ethics Board, Lund, Sweden, in order to protect people’s privacy. Data are available from Disa Dahlman, disa.dahlman@med.lu.se, for researchers who meet the criteria for access to confidential data.

## Abbreviations

AOR: Adjusted Odds ratio
CI: Confidence interval
COPD: Chronic obstructive pulmonary disease
ED: Emergency department
NEP: Needle exchange program
OR: Odds ratio
OST: Opioid substitution treatment
OUD: Opioid use disorder
PHC: Primary healthcare
SUD: Substance use disorders
